# A System of Practical Medicine by American Authors

**Published:** 1899-03

**Authors:** 


**A System of Practical Medicine by American Authors.** Edited by Alfred Lee Loomis, M.D., and William Gilman Thompson, M.D. Vol. III.—*Diseases of the Alimentary Canal —Diseases of the Peritoneum—Diseases of the Liver and Gall Bladder—Diseases of the Spleen—Diseases of the Pancreas—Diseases of the Thyroid Gland—Chronic Metal-Poisoning—Alcoholism; Morphinism, etc.* Pp. 5—9, 19—926. London: Henry Kimpton. 1898.

The list of contributors to this volume comprises nine physicians of New York State, three of Canada, one of Boston, two of Ann Arbor, one of Chicago, one of San Francisco, one of Washington, and one of Philadelphia; a list which may be looked upon as fairly representative of the North American continent.

Dr. Stockton and Dr. Allen Jones, in their article on diseases of the stomach, give an account of what can be done which is much in advance of the ordinary text-books. Their account of the general examination of the patient and the physical examination of the stomach comprises the use of all modern methods, and their account of the general and chemical analysis of the stomach-contents is especially full and complete. The subjects of hyperchlorhydria, hypochlorhydria, and achylia gastrica are described fully, and the use of the gastric electrode is advocated, both for the correction of defective secretion and in the treatment of the gastric sensory and motor neuroses. In the treatment of gastric ulcer the authors advocate the use of a combination of cerium oxalate, bismuth subcarbonate, and the light magnesium carbonate in large and oft repeated doses, and they do not advise rectal feeding excepting for at least three days after a gastric hemorrhage. It is somewhat of a surprise that they do not peptonise the milk for rectal feeding, and altogether do not give the practice of pre-digestion of milk the attention which its utility appears to demand. In the diagnosis of gastric carcinoma the use of Einhorn's gastrodiaphane is used to "assist in determining the size and location of the neoplasm, as the transillumination shows with less intensity or not at all through the carcinomatous anterior wall of the stomach in contrast to the brighter light transmitted through the rest of the organ." The chapter on gastrectasia and gastroptosis is very complete, and Einhorn's double tube gastric vaporizer has been found to act as an analgesic and slight motor stimulant. The authors emphasise the importance of surgical treatment of pyloric stenosis, especially of the benign kind, in which pylorectomy has been successful in 54 per cent., gastro-enterostomy in 71 per cent., pyloroplasty in 77.4 per cent., and digital divulsion in 59 per cent.

In addition to the topics named at the head of this review, the volume deals with infectious diseases common to man and animals, and with purpura, beri-beri, hæmophilia, diabetes, and insolation.

